# Down-regulation of *Fusarium oxysporum* endogenous genes by Host-Delivered RNA interference enhances disease resistance

**DOI:** 10.3389/fchem.2015.00001

**Published:** 2015-01-20

**Authors:** Zongli Hu, Urvi Parekh, Natsumi Maruta, Yuri Trusov, Jose R. Botella

**Affiliations:** ^1^Plant Genetic Engineering Laboratory, School of Agriculture and Food Sciences, University of QueenslandBrisbane, QLD, Australia; ^2^Bioengineering College, Chongqing UniversityChongqing, China

**Keywords:** Host-Delivered RNAi, host-induced gene silencing, *Fusarium oxysporum*, disease resistance, disease control, plant fungal pathogens

## Abstract

*Fusarium oxysporum* is a devastating pathogen causing extensive yield losses in a variety of crops and development of sustainable, environmentally friendly methods to improve crop resistance is crucial. We have used Host-Delivered RNA interference (HD-RNAi) technology to partially silence three different genes (*FOW2, FRP1*, and *OPR*) in the hemi-biotrophic fungus *F. oxysporum* f. sp. *conglutinans*. Expression of double stranded RNA (dsRNA) molecules targeting fungal pathogen genes was achieved in a number of transgenic Arabidopsis lines. *F. oxysporum* infecting the transgenic lines displayed substantially reduced mRNA levels on all three targeted genes, with an average of 75, 83, and 72% reduction for *FOW2, FRP1*, and *OPR*, respectively. The silencing of pathogen genes had a clear positive effect on the ability of the transgenic lines to fight infection. All transgenic lines displayed enhanced resistance to *F. oxysporum* with delayed disease symptom development, especially *FRP1* and *OPR* lines. Survival rates after fungal infection were higher in the transgenic lines compared to control wild type plants which consistently showed survival rates of 10%, with *FOW2* lines showing 25% survival; *FRP1* lines 30–50% survival and *OPR* between 45 and 70% survival. The down-regulation effect was specific for the targeted genes without unintended effects in related genes. In addition to producing resistant crops, HD-RNAi can provide a useful tool to rapidly screen candidate fungal pathogenicity genes without the need to produce fungal knockout mutants.

## Introduction

The genus *Fusarium* includes several species of fungi that are broadly spread in soil and organic substrates worldwide. *Fusarium oxysporum* is one of the most relevant species of this genus and is the causal agent of root rots, damping-off and wilt diseases in more than 100 plants species, including a wide range of economically important horticultural crops, flowers, trees, and a number of field crops such as cabbage, banana, and cotton (Michielse and Rep, 2009; Takken and Rep, 2010; Dean et al., 2012; Swarupa et al., 2014). *F. oxysporum* consists of over 120 *forma specialis* (f. sp.) of pathogenic strains determined by their primary host plants (Armstrong and Armstrong, 1981; Di Pietro et al., 2003; Fourie et al., 2011). All strains of *F. oxysporum* are saprophytic, being able to grow and survive for long periods on organic matter in soil making it very difficult to control (Olivain and Alabouvette, 1997). Its pathogenic life cycle starts with spore germination upon recognition of a suitable host; once the hyphae is formed, the pathogen enters its host by directly penetrating the roots and colonizes it within the xylem by producing microconidia which leads to mycelia formation (Di Pietro et al., 2003). Colonization and toxin production by the pathogen results in blockage of the host vascular system (Michielse and Rep, 2009), causing characteristic disease symptoms including vasculature yellowing, vein clearing, chlorosis, and necrosis in leaf veins and leaves, leaf detachment and wilting (Di Pietro et al., 2003; Czymmek et al., 2007). After the plant dies, the fungus sporulates on the decayed leaf surfaces. *F. oxysporum* is most prevalent in tropical and subtropical regions and it is expected that its geographical range will extend due to climate change (Okubara and Paulitz, 2005).

Current control methods for Fusarium wilt are very limited with crop rotations being ineffective due to the large host range and its persistence in soil (Davis et al., 2006). Management of Fusarium wilt is mainly done through cultural practices and farm hygiene which only reduce the transmission of inoculum while soil sterilization can only be performed in glasshouses (Michielse and Rep, 2009). Soil fumigation using broad-spectrum biocides such as methyl bromide is expensive and has many hazardous effects on the environment (Fravel et al., 2003; Davis et al., 2006). Natural resistance on a gene for gene relationship between hosts and *F. oxysporum* races has been described and used to develop resistant crop varieties providing an environmentally safe control method (Roncero et al., 2003; Michielse and Rep, 2009). However, breeding for resistance is not always an easy process and new races of the pathogen can develop to overcome host resistance (Fravel et al., 2003).

RNA interference (RNAi) is a cellular process found in most eukaryotes involved in developmental regulation of gene expression as well as defense against viruses and transposons (Fire et al., 1998; Watson et al., 2005; Mahmood-Ur-Rahman et al., 2008; Jinek and Doudna, 2009; Obbard et al., 2009). RNAi is triggered by the presence of double stranded RNA (dsRNA) in the cell and ultimately leads to the degradation of homologous single stranded RNA molecules, inhibition of translation and modification of homologous genomic sequences (Qi and Hannon, 2005; Mahmood-Ur-Rahman et al., 2008). The artificial manipulation of this pathway has allowed the efficient silencing of genes in transgenic plants and provided a powerful platform for reverse genetics studies (Duan et al., 2012). An alternative approach involving RNAi, known as Host-Delivered RNAi (HD-RNAi) or Host Induced Gene Silencing (HIGS), has emerged in recent years that use the host plant as a delivery system to cause gene silencing in the pathogen (Fairbairn et al., 2007; Mahmood-Ur-Rahman et al., 2008). In the HD-RNAi approach, the host plant is transformed with a hairpin construct targeting a pathogen gene. Once the transgenic plant is infected and the pathogen starts feeding from the host, the small interfering RNA (siRNA) molecules are transferred to the pathogen cells, ultimately inducing an RNAi response in the pathogen and the silencing of the targeted gene.

HD-RNAi have been proven to work on many plant pathogens such as root knot nematodes where the silencing observed in nematodes feeding from transgenic plants expressing RNAi constructs was specific for the targeted genes with no off-target effects and, in some cases, the transgenic plants were resistant to nematode colonization (Huang et al., 2006; Fairbairn et al., 2007; Dubreuil et al., 2009). RNAi has been used to confer resistance against bacterial pathogens, including crown gall disease caused by *Agrobacterium tumefaciens* (Escobar et al., 2001, 2002; Escobar and Dandekar, 2003; Viss et al., 2003). HD-RNAi is also effective in plant-insect systems and has been used to effectively silence genes in cotton bollworm larvae (*Helicoverpa armigera*) and down-regulation of the *V-ATPase A* gene in western corn rootworm (*Diabrotica virgifera virgifera*) larvae resulted in reduced damage to cotton plants (Baum et al., 2007; Mao et al., 2007). HD-RNAi has been successfully used to silence genes in parasite weeds providing partial protection to the host plant (Tomilov et al., 2008; Aly et al., 2009). Curiously, the RNAi effect can go both ways, as RNAi signals have also been proven to travel from the parasitic plant to the host (Tomilov et al., 2008). On the other hand, HD-RNAi has been proven ineffective in the control of *Striga asiatica*, an economically important parasite of maize (de Framond et al., 2007). The application of HD-RNAi to fungal pathogens is quite recent and has used a number of different approaches to produce dsRNA molecules in the host plant. Mixed success to provide disease resistance against the rust fungi *Puccinia striiformis* and *Puccinia triticina* using the virus-induced gene silencing (VIGS) technique has been reported (Yin et al., 2011; Panwar et al., 2013a,b). Particle bombardment of hairpin constructs to barley and VIGS in wheat resulted in down-regulation of genes in the powdery mildew *Blumeria graminis* (Nowara et al., 2010). Efforts to use HD-RNAi in oomycetes have been so far unsuccessful (Zhang et al., 2011). Furthermore, a recent report has measured the *in vitro* antifungal activities of a set of synthetic dsRNAs applied to fungal spores of *F. oxysporum* f. sp. *cubense* and *Mycosphaerella fijiensis*, the causing agents of Fusarium wilt and black sigatoka in bananas, respectively (Mumbanza et al., 2013). The results are promising and indicate that some of the synthetic dsRNAs resulted in inhibition of spore germination. HD-RNAi has been recently used on several *Fusarium* species. *GUS* gene expression in *F. verticillioides* was significantly suppressed after feeding on transgenic tobacco plants expressing *GUS*-RNAi (Tinoco et al., 2010). More recently, HD-RNAi approach was also tested in Arabidopsis and barley against *F. graminearum*. It was demonstrated that HD-RNAi effectively silenced three fungal cytochrome P450 lanosterol C-14 α-demethylase (CYP51) genes and resulted in strongly enhanced resistance to the pathogen (Koch et al., 2013).

*F. oxysporum* (f. sp. *conglutinans*) infects the family *Brassicaceae* including Arabidopsis. It is known that in Arabidopsis, *F. oxysporum* enters the host through the primary root tip and lateral root emerging points (Di Pietro et al., 2001; Czymmek et al., 2007). Normally 3–4-week-old Arabidopsis plants develop characteristic chlorosis along the leaf veins 5–7 days after inoculation (Trusov et al., 2013). The susceptible genotypes decay approximately 20 days after inoculation, and distinguished fungal sporulation occurs on the plant surfaces. There are obvious advantages in using this particular host-pathogen interaction system. Firstly, simple inoculation assays that resemble natural infection process starting from roots progressing to vasculature have been developed (Trusov et al., 2013). Secondly, this system allows quantification of disease symptoms, as the number of leaves showing chlorotic veins per plant can be recorded throughout the plant disease progression (Trusov et al., 2013). Finally, aside from symptom quantification, the survival percentage by the number of plants which has thrived 15 days after inoculation can also be counted (Trusov et al., 2013). Here, we have produced transgenic Arabidopsis lines expressing hairpin constructs targeting three different *F. oxysporum* genes and shown down-regulation of all targeted genes in fungus infecting the transgenic lines. The effect of the silencing on disease progression and ultimate survival of the plants is evaluated and effectiveness of HD-RNAi technology in practice is discussed.

## Materials and methods

### Pathogen preparation and inoculations

*F. oxysporum* (f. sp *conglutinans*) (BRIP 5176; Department of Primary Industries, Queensland, Australia) were grown and plants were inoculated as previously described (Trusov et al., 2006, 2013). In summary, plants were dipped into the fungal inoculum for 30 s after carefully cleaning the roots with water. *Fusarium* inoculated plants were replanted into fresh soil and grown at 28°C in a growth cabinet with required humidity and light intensity. Twenty plants from each transgenic line as well as wild type were inoculated. The degree of infection was measured as symptoms appeared and progressed in a time span of 7–12 days post inoculation (dpi), by counting the number of leaves with yellow veins. The total number of leaves was also counted and found to be the same in all genotypes. The percentage of surviving plants out of 20 plants was recorded after a minimum of 15 dpi. Three infection experiments were performed for each gene with similar results. Fungal RNA was extracted from the aerial part of Arabidopsis plants 7 days after inoculation as described previously (Chakravorty et al., 2011).

### Construction of plasmid and plant transformation

The *F-box protein Required for Pathogenicity 1 (FRP1)* (Genbank AY673970.1), *F. oxysporum Wilt 2* (*FOW2*) (GenBank AB266616.1) and a previously uncharacterized gene with homology to plant 12-oxophytodienoate-10,11-reductase gene (*OPR*) (Genbank AFQF01002613) fragments used for the construction of the RNAi constructs were amplified from *F. oxysporum* cDNA by PCR using the primers shown in Table 1.

**Table 1:** **List of primer sequences for cloning RNAi constructs**.

*FRP1-F*	5′-GATCTAGACTCGAGACTTGCCTCCAAATCGTG-3′
*FRP1-R*	5′-GAGGATCCGAATTCTCTATTGAGCCAGAACTCC-3′
*FOW2-F*	5′-GCTCTAGACTCGAGAAGTCTGGCTCTAGTGGAAA-3′
*FOW2-R*	5′-CTGGATCCGAATTCATCTGTTGGGTCGCTATT-3′
*OPR-F*	5′-AAAGCTTGGTACCAGAAACCGAGGAACTCCGG-3′
*OPR-R*	5′-TTCTAGAATTCAGTTCACGACAGAGGTGACTC-3′

*Restriction enzyme recognition sequences are underlined*.

The *FRP1* and *FOW2* fragments were 817 and 846 bp, respectively and were cloned into the pHannibal intermediate RNAi vector in the sense (*Xho*I/*Eco*RI) and antisense (*Bam*HI/*Xba*I) orientations under the control of the cauliflower mosaic virus *35S* and the *OCS*-terminator. The RNAi cassettes were then excised with *Not*I and cloned into the pUQC247 binary vector. In the case of *OPR*, the fragment was 746 bp long and was cloned into the pKannibal intermediate RNAi vector using *Hind*III/*Xba*I and *Eco*RI/*Kpn*I for the sense and antisense fragments, respectively and the *Not*I fragment was later transferred to the pART27 binary vector. The RNAi plasmids were transferred to *Agrobacterium tumefaceins* by triparental mating (Vanhaute et al., 1983), and transgenic Arabidopsis plants (Columbia-0 ecotype) generated by *Agrobacterium*—mediated transformation was done using floral dipping (Clough and Bent, 1998). All experiments were carried out using homozygous T3 lines.

### Quantitative real-time RT-PCR analysis

Fungal RNA was extracted from the aerial part of Arabidopsis plants 7 days after inoculation as described previously (Purnell and Botella, 2007). Quantitative Real Time- (qRT)-PCR was performed as described by Koia et al. (2012). In summary, first strand cDNA synthesis was conducted using the SuperScript III RT kit (Invitrogen) according to the manufacturer's instructions. qRT-PCR was performed using Power SYBR Green PCR Master Mix (Applied Biosystems) and the 7900 HT Sequence Detection System (Applied Biosystems). The primer pairs used for qRT-PCR are shown in Table 2 and were designed using Primer Express software (Applied Biosystems).

**Table 2:** **List of primer sequences for qRT-PCR**.

**Gene**	**Forward**	**Reverse**
*FRP1*	5′-ATCAGCGTCAACCTCCTCCGACT-3′	5′-ATGGTCAAGGGGCCTTAGAGGT-3′
*FOW2*	5′-TCCAGTCCCAGCTCTGGATCC-3′	5′-AGGTCCAGTGGATGAGCGCCT-3′
*OPR*	5′-TCGTTGAACGGCGGTATGAGCA-3′	5′-ACATCATTGAATCCGCCAGCGGA-3′
*ACTIN*	5′-CACCACCTTCAACTCCATCA-3′	5′-TCGGAGAGACCAGGGTACAT-3′

Gene expression analysis was performed using SDS Version 2.2.2 software (Applied Biosystems). The results shown are average values from three independently prepared RNA samples.

## Results

### Selection of fungal genes for down regulation and production of transgenic lines

The purpose of our research was to determine whether the HD-RNAi technology can be used to silence genes in *F. oxysporum*. Although many pathogen genes would be adequate targets for this purpose, the ultimate application of the technology is to produce plants resistant to the disease and it is therefore important to identify genes required for fungal pathogenicity. Forward genetics studies have revealed a number of genes involved in *Fusarium* pathogenicity including argininosuccinate lyase (*ARG1*); class V and class VII chitin synthases (*CHSV* and *CHSVb*); two different heterotrimeric G protein α subunits (*FGA1* and *FGA2*); a heterotrimeric G protein β subunit (*FGB1*), a MAP kinase (*FMK1*) and a mitochondrial carrier protein (*FOW1*) (Di Pietro et al., 2001; Namiki et al., 2001; Inoue et al., 2002; Jain et al., 2002, 2003, 2005; Madrid et al., 2003; Martin-Urdiroz et al., 2008).

For this study we selected three *Fusarium* genes with different characteristics. The first targeted gene, *FOW2* (Genbank AB266616.1) encodes a putative transcription regulator belonging to the Zn(II)2Cys6 family and has been shown to be essential for pathogenicity of *F. oxysporum* f. sp. *melonis* (Imazaki et al., 2007). *FOW2*-targeted mutants completely lost pathogenicity, being unable to colonize the roots of the host but were not impaired in vegetative growth or conidiation when grown in culture. In addition, mutations in the same gene of a different *forma specialis* (f. sp. *lycopersici*) also resulted in loss of pathogenicity, implying the universal importance of the gene for pathogenicity. The second gene selected was *FRP1* (Genbank AY673970.1) which encodes an F-box protein that interacts with SKP1 to facilitate targeting of proteins to the SCF-ubiquitination complex. FRP1 is necessary for *F. oxysporum* f. sp. *lycopercisi* pathogenicity and, as with FOW2, FRP1-deficient mutants were still able to grow in artificial media but did not colonize host plants (Duyvesteijn et al., 2005). The final target of our study is a gene encoding for a protein with homology to 12-oxo-phytodienoate reductase (OPR) (Genbank AFQF01002613), an enzyme involved in the biosynthesis of jasmonic acid (JA) in plants (Wasternack, 2007). It has been recently shown that JA-insensitive *coi1* Arabidopsis mutants display nearly complete resistance to *F. oxysporum* f. sp. *conglutinans* and it is believed that *F. oxysporum* might produce JA and use it to target the COI1 protein and to disrupt the host's defense mechanism (Thatcher et al., 2009; Trusov et al., 2009). The JA biosynthetic pathway has not been established in fungi but Genebank searches using the translated Arabidopsis At2g06050 gene (encoding for OPR3) found a homologous gene in chromosome 4 of *F. oxysporum* (Fo5176), which is a variant of f. sp. *conglutinans*, and hereafter we call the gene *OPR*.

A fragment of ~750–850 bp for each of the selected genes was cloned into an intermediate vector in sense and antisense orientations with the PDK intron as a spacer under the control of the Cauliflower mosaic virus 35S promoter (CaMV 35S) and the RNAi cassette was excised and cloned into a binary vector (Wesley et al., 2001) (Supplementary Figure S1). In order to avoid the chances of cross effects of the ectopic siRNAs on plant transcripts in our HD-RNAi studies we selected cDNA fragments with little or no homology with their plant variants. The *F. oxysporum FOW2, FRP1*, and *OPR* fragments used in our study were used in BLAST searches against the fully sequenced Arabidopsis genome. No homologous sequences for *FOW2* or *FRP1* were found in Arabidopsis neither using high or low stringency parameters. For the *OPR* fragment an overall sequence identity of 43% was found between the fungal and Arabidopsis counterparts. Wild type Arabidopsis ecotype Columbia-0 (Col-0) plants were transformed with the specific RNAi constructs for each of the three targeted genes using *A. tumefaciens* (LBA4404). Ten to fifteen independent transgenic lines were generated for each of the three constructs and 2–3 stable homozygous lines were selected and further characterized. Although RNAi constructs are specifically designed to target gene expression in *F. oxysporum*, it is plausible that off-target effects may occur. We searched the entire Arabidopsis genome for genes with homology to either *FOW2* or *FRP1*, however, we did not find highly similar sequences (data not shown). We also compared the sequences of the Arabidopsis *OPR3* and *F. oxysporum OPR* genes. Despite the evident homology at the protein level, the DNA sequences were displayed divergence low level of similarity (43% overall nucleotide identity) (Supplementary Figure S2) preventing a possibility for off-target silencing in transgenic Arabidopsis lines. The resulting Arabidopsis transgenic lines for each *FOW2*-, *FRP1*-, and *OPR*-RNAi did not display any observable phenotypic differences from Col-0 wild type plants in germination, growth parameters and seed production. Importantly, Arabidopsis mutants lacking OPR3 (*opr3*) are male-sterile; and therefore unable to produce seeds by self-pollination (Stintzi and Browse, 2000). We produced over 30 Arabidopsis *OPR*-RNAi transgenic lines, all of which produced WT looking siliques and subsequently seeds, therefore confirming that there were no off-target gene silencing effects in the Arabidopsis HD-RNAi transgenic lines.

### HD-RNAi results in down-regulation of targeted genes in *F. oxysporum* infecting transgenic Arabidopsis lines

To determine whether HD-RNAi results in down-regulation of the targeted *F. oxysporum* genes we measured the relative expression levels for all three genes in fungi infecting their corresponding transgenic lines and compared them with the levels present in fungi infecting wild type Arabidopsis plants. For this purpose, total RNA was extracted from above-ground tissues of infected plants 7 days after inoculation with the pathogen. At this stage, fungal mycelium is abundant in the vasculature of the host plant leaves and stems. Fungal gene expression levels were determined by quantitative real time PCR (qRT-PCR) with gene-specific primers designed to bind outside the DNA fragments used for the RNAi constructs to avoid possible artifacts. Levels of *F. oxysporum* actin (GenBank JQ965663.1) were assumed to remain constant in all fungal cells and were therefore used for normalization of results. Optimization experiments showed that all primer pairs were able to amplify their intended specific targets, confirmed by sequencing, with reproducible Ct values (results not shown).

**Figure 1:**
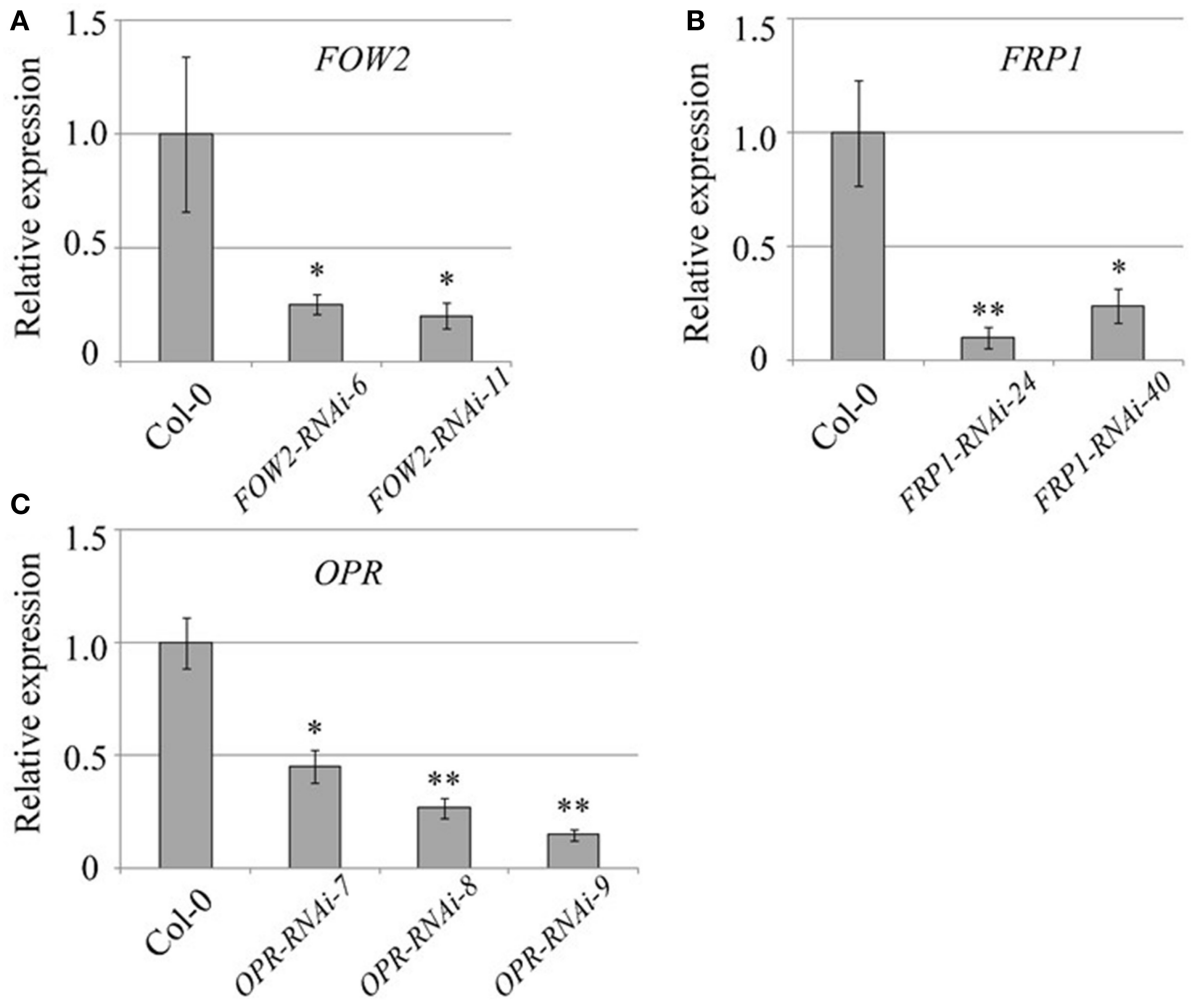
**Quantification of *Fusarium oxysporum* mRNA levels**. Wild type and T3 homozygous transgenic lines expressing the HD-RNAi constructs for **(A)** *FOW2*, **(B)** *FRP1*, and **(C)** *OPR* were infected with *F. oxysporum* f. sp. *conglutinans*. Total RNA extracted from above-ground tissues of 3-week-old transgenic lines at 7 days post inoculation was used (*n* = 20) for cDNA synthesis as a template for quantitative real time PCR with pathogen gene-specific primers. *F. oxysporum* actin levels (GenBank JQ965663.1) were used for normalization purposes. For each gene, the relative mRNA level measured in *F. oxysporum* infecting wild type plants was given the arbitrary value of one and the remaining mRNA levels referred to it. Values shown are means ± SE of three biological replicates. ^*^p ≤ 0.05; ^**^p ≤ 0.005.

Figure 1 shows that for each of the three targeted genes, the mRNA levels in fungi infecting their respective Arabidopsis HD-RNAi lines are lower than those observed in fungi infecting Col-0 controls. *FOW2* mRNA levels were reduced by 75 and 78% in the two independent transgenic lines assayed (Figure 1A). Meanwhile, *FRP1* levels were reduced by 90 and 76% in the two transgenic lines assayed (Figure 1B). Down-regulation of *OPR* expression levels was variable with a reduction in mRNA levels of 59, 75, and 83% in the three transgenic lines studied (Figure 1C). It is important to remark that, even though in some cases the reduction in mRNA levels was quite dramatic, no complete silencing was achieved for any of the targeted genes.

### Disease progression in HD-RNAi transgenic Arabidopsis lines

**Figure 2:**
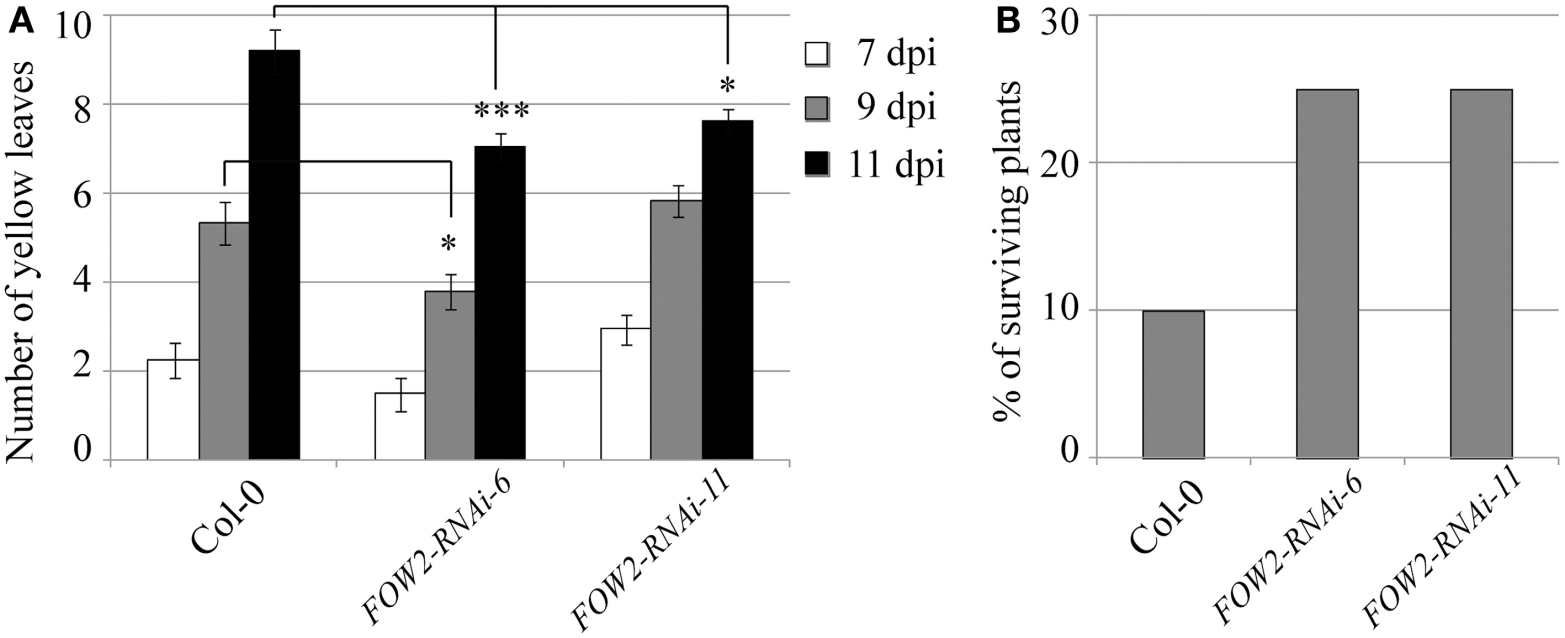
**Disease progression of transgenic *FOW2*-RNAi lines**. Two independent T3 homozygous Arabidopsis transgenic lines carrying an HD-RNAi construct targeting the *F. oxysporum FOW2* gene were infected with *F. oxysporum*. **(A)** Disease progression was monitored by quantifying the number of yellow leaves. Values shown are means ± SE (*n* = 20). The experiment was repeated three times. **(B)** Survival was assessed 2–4 weeks after infection. Plants surviving after 2 weeks would grow to maturity and set seeds. Wild type Col-0 plants were used as control. A representative of three experiments is shown. ^*^p ≤ 0.05; ^***^p ≤ 0.0005.

Even though *FOW2* mRNA levels in fungi infecting transgenic FOW2 HD-RNAi lines was reduced to 22–28% of the normal expression levels detected in fungi infecting wild type plants, the effect on disease progression measured by the number of yellow (chlorotic) leaves per plant was not as dramatic (Figure 2A). Only one transgenic line, *FOW2*-RNAi-6, showed a consistent and statistically significant decrease in yellow leaves counts at the advanced infection stages, indicating a slow-down in the disease progression (Figure 2A). Nevertheless, although the disease progression was not radically slowed, the percentage of surviving plants at 18 days-post inoculation was significantly improved in both transgenic lines (*FOW2*-RNAi-6 and *FOW2*-RNAi-11) to more than double of number observed in wild type plants (Figure 2B).

**Figure 3:**
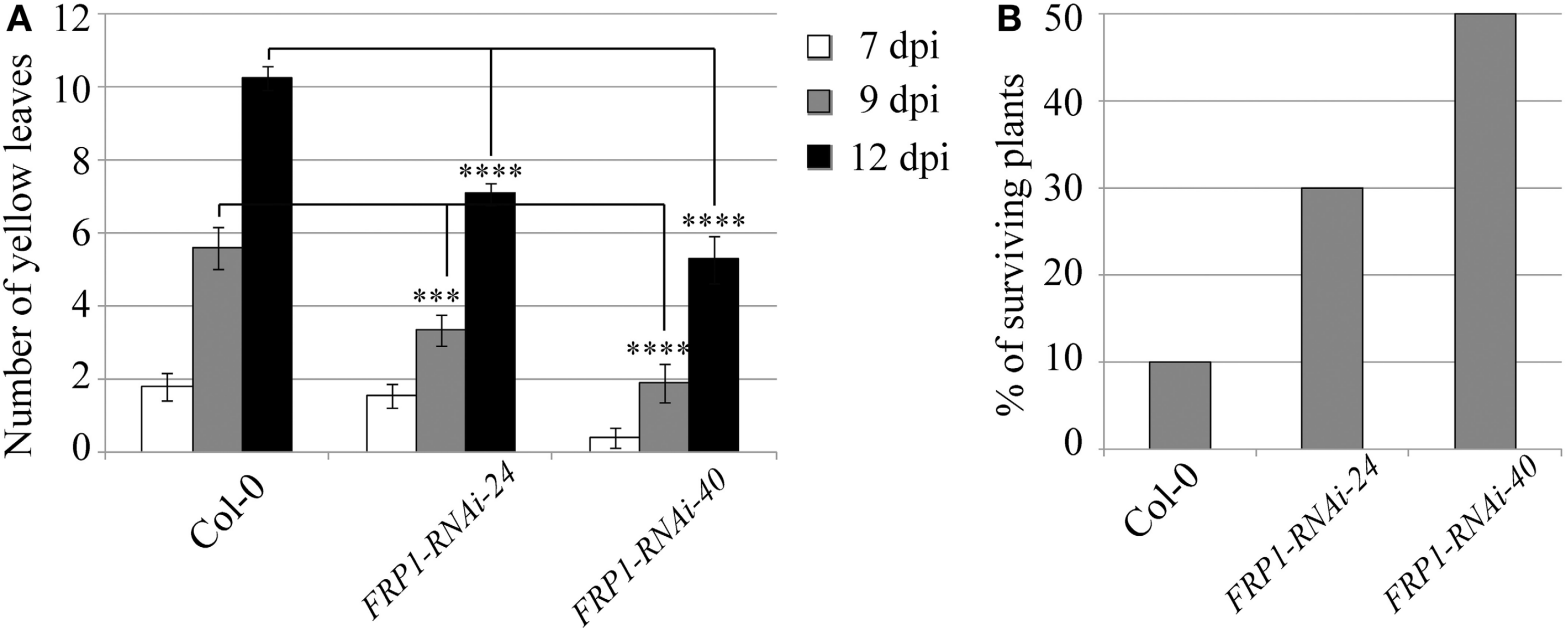
**Disease progression of transgenic *FRP1*-RNAi lines**. Two independent T3 homozygous Arabidopsis transgenic lines carrying an HD-RNAi construct targeting the *F. oxysporum FRP1* gene were infected with *F. oxysporum*. **(A)** Disease progression was monitored by quantifying the number of yellow leaves. Values shown are means ± SE (*n* = 20). The experiment was repeated three times. **(B)** Survival was assessed 2–4 weeks after infection. Plants surviving after 2 weeks would grow to maturity and set seeds. Wild type Col-0 plants were used as control. A representative of three experiments is shown. ^***^p ≤ 0.0005; ^****^p ≤ 0.0001.

A very different behavior was observed in the transgenic lines targeting the fungal *FRP1* gene. Disease progression was clearly slowed down in the transgenic lines compared to wild type controls, with an statistically significant reduction in the number of yellow leaves observed for both transgenic lines at the advanced stages of infection (Figure 3A). As a consequence, survival rates were greatly enhanced in the transgenic lines with 30 and 50% surviving fungal infection in lines *FRP1*-RNAi-24 and *FRP1*-RNAi-40 respectively, compared to 10% in wild type plants (Figure 3B).

**Figure 4:**
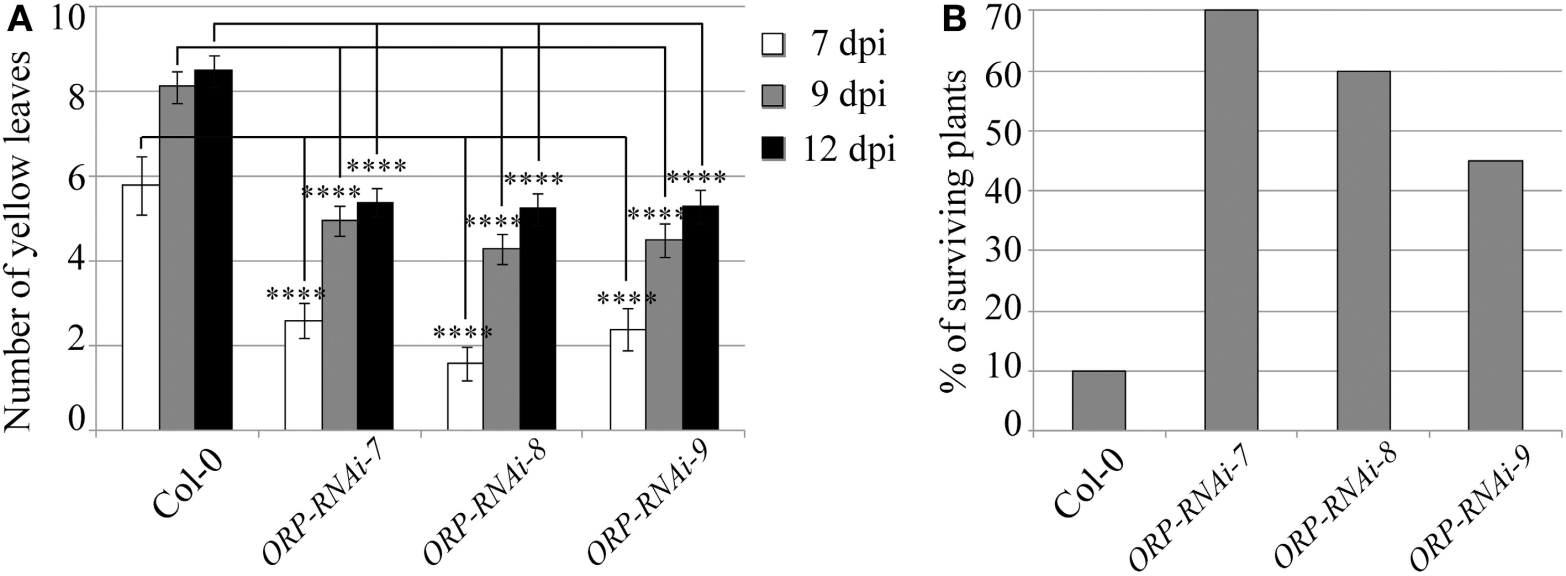
**Disease progression of transgenic *OPR*-RNAi lines**. Three independent T3 homozygous Arabidopsis transgenic lines carrying an HD-RNAi construct targeting the *F. oxysporum OPR* gene were infected with *F. oxysporum*. **(A)** Disease progression was monitored by quantifying the number of yellow leaves. Values shown are means ± SE (*n* = 20). The experiment was repeated three times. **(B)** Survival was assessed 2–4 weeks after infection. Plants surviving after 2 weeks would grow to maturity and set seeds. Wild type Col-0 plants were used as control. A representative of three experiments is shown. ^****^p ≤ 0.0001.

**Figure 5:**
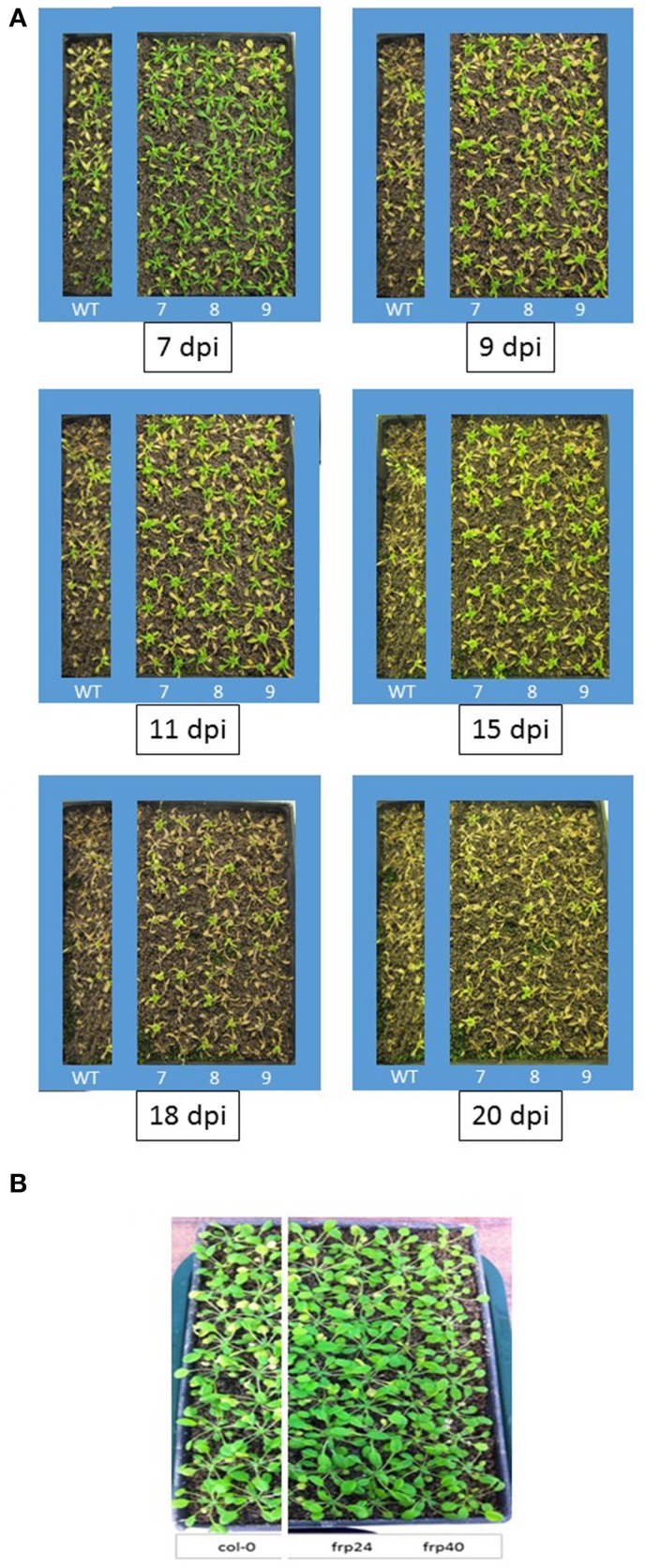
**Time-lapse images and disease progression in transgenic *OPR*-RNAi and *FRP1*-RNAi lines**. **(A)** Three independent T3 homozygous Arabidopsis OPR transgenic lines (#7, #8, and #9) carrying an HD-RNAi construct targeting the *F. oxysporum OPR* gene were infected with *F. oxysporum*. Disease progression was photographed 7, 9, 11, 15, 18, and 20 days after inoculation. Plants surviving after 2 weeks would grow to maturity and set seeds. Wild type Col-0 plants were used as control. A representative of three experiments is shown. **(B)** Two independent T3 homozygous Arabidopsis FRP1 transgenic lines (#24 and #40) carrying an HD-RNAi construct targeting the *F. oxysporum FRP1* gene were infected with *F. oxysporum*. Disease progression was photographed 9 days after inoculation.

Targeting of the *F. oxysporum OPR* gene produced the best results in terms of disease symptom progression with all three studied transgenic lines (*OPR*-RNAi-7, *OPR*-RNAi-8, and *OPR*-RNAi-9) producing significantly lower numbers of yellow leaves than wild type plants from the very early stages of infection (Figure 4A). In addition, the survival rates were quite high with 70, 60, and 45% for transgenic lines *OPR*-RNAi-7 and *OPR*-RNAi-8 and *OPR*-RNAi-9 respectively compared to the 10% survival rate for wild type controls (Figure 4B). A complete time-lapse sequence showing the disease development in the three transgenic OPR lines is shown in Figure 5. Plants surviving infection after 20 days would produce flowers and set seeds.

## Discussion

In this work, we have shown that expression of dsRNA molecules targeting pathogen genes in transgenic Arabidopsis plants can effectively reduce the mRNA levels of three different genes in *F. oxysporum* infecting the transgenic lines. The level of down regulation achieved in the different lines was substantial, with an average of 75, 83, and 72% for *FOW2, FRP1*, and *OPR*, respectively. Two selected transgenic *FOW2*-RNAi lines displayed elevated levels of resistance against *F. oxysporum* infection with slowed disease progression and improved survival rates compared to wild type controls (Figures 2A,B). Two *FRP1*-RNAi transgenic lines as well showed clearly delayed disease symptom development as compared to wild type and as expected it resulted in the enhanced survival rate (Figures 3A,B). The expression level of *F. oxysporum OPR* gene was more variable between transgenic *OPR*-RNAi lines it fed on, perhaps due to the fact that the normal expression levels were quite low compared to the other two genes, hence getting closer to the qRT-PCR detection limit. However, all three transgenic *OPR*-RNAi lines tested were evidently more resistant to *F. oxysporum* than wild type plants, indicated from the reduced yellow leaf symptoms in all the transgenic lines and markedly increased survival (Figures 4A,B).

FOW2 has been proven to be important for pathogenicity in two different *F. oxysporum* forma specialis, f. sp. *melonis* and f. sp. *lycopersici* and it has been suggested that it encodes a transcription regulator controlling the infection competency of *F. oxysporum* pathogens (Imazaki et al., 2007). FOW2-defective mutants completely lost their ability to invade plant roots and colonize the plant, even when the pathogen was manually injected into the plant tissues. The fact that we observe relatively mild, although statistically significant, differences in disease progression between the wild type control and the transgenic *FOW2*-RNAi lines might be due to the incomplete silencing achieved in the fungi. mRNA levels were reduced by 75 and 78% in transgenic lines but it is possible that the residual ~25% is enough for *FOW2* to confer pathogenicity, and thus stronger silencing is needed in order to observe a more prominent impact on disease progression. An alternative explanation is that *FOW2* might function largely at the onset of the infection process, perhaps involved in detection of the potential host, while RNAi silencing can only occur after the fungus is established in the host plant. Therefore, the silencing might come a bit late to exert a full impact on the pathogen. Nevertheless, the substantial increase in survival rates observed in both transgenic lines, suggests that the levels of down-regulation achieved for *FOW2* do have an effect on the ability of the pathogen to colonize the plant.

FRP1 is essential for pathogenicity in *F. oxysporum* f. sp. *lycopersici* and it is highly conserved in f. sp. *conglutinans*. Disruption in *FRP1* led to a complete loss of pathogenicity in tomato with mutant fungi unable to colonize the roots (Duyvesteijn et al., 2005). The fact that FRP1 is an F-box protein and that it physically interacts with *F. oxysporum* SKP1 (Genebank AAT85970.1) suggests that FRP1 is part of a SCF ubiquitin kinase complex. The *F. oxysporum* f. sp. *lycopersici* FRP1 protein has highly homologous counterparts (86–100% identity) in other *Fusarium* species (*F. oxysporum* f. sp. *conglutinans*; f. sp. *cubense* races 1 and 4, *F. fujikuroi, F. graminearum* and *F. pseudograminearum*) and it is also present in other plant pathogenic fungi such as *Colletotrichum graminicola* (63% identity) and even non plant pathogenic fungi such as *Beauveria bassiana* (66% identity) and *Neurospora crassa* (55% identity). The relatively high level of homology is probably due to the conservation of the F-box motif and points to the suggestion that an FRP1-associated SCF ubiquitin ligase complex plays an important role in degradation of pathogenicity-related proteins through ubiquitination (Duyvesteijn et al., 2005). Even though it has been suggested that FRP1 plays a crucial role in the early events of the Fusarium infection process, in our hands, partial silencing of the *FRP1* gene in fungi infecting transgenic Arabidopsis *FRP*-RNAi lines led to a statistically significant delay in disease symptoms and a marked increase in survival rates. Therefore, in addition to its importance during the first steps of infection, FRP1 must have additional role/s in the colonization of the plant tissue.

JA is a well-known phytohormone involved in defense and is particularly important against necrotrophic pathogens (Nickstadt et al., 2004; Glazebrook, 2005). Studies in Arabidopsis have shown that overexpression of defense-related proteins in the JA pathway, such as THI2.1, ERF1, and ERF2, confer enhanced resistance to *F. oxysporum* (Epple et al., 1997; McGrath et al., 2005; Berrocal-Lobo and Molina, 2008). In addition, T-DNA knockouts of AtMYC2, a negative regulator of JA-ethylene defense responsive genes also results in enhanced *F. oxysporum* resistance (Anderson et al., 2004; Trusov et al., 2009; Trusov and Botella, 2012), suggesting that the JA-mediated pathway plays a defense role against *F. oxysporum*. Strikingly, it was recently discovered that mutants lacking the JA receptor COI1, display increased resistance to *F. oxysporum* while mutants defective in JA biosynthesis displayed wild type levels of resistance (Thatcher et al., 2009; Trusov et al., 2009). These results indicate that even though JA generally mediates immunity, it is highly possible that the COI1-mediated signaling pathway contributes to susceptibility to *F. oxysporum* (Thatcher et al., 2009; Trusov et al., 2009). Notably, it has been established that fungi also produce JA and various other jasmonates with over 20 jasmonate compounds identified in *F. oxysporum f. sp. matthiolae* (Miersch et al., 1999; Wasternack, 2007). It has been therefore hypothesized that *F. oxysporum* might use its own JA to target COI1 and hijack the host's defense mechanism (Thatcher et al., 2009).

In plants JA is synthesized from α-linolenic acid in a number of reactions catalyzed by lipoxygenases (LOXs), allene oxide synthases (AOSs), and allene oxide cyclases (AOCs) to form 12-oxo-phytodienoic acid (OPDA), which is then converted to JA via 12-oxo-phytodienoate reductases (OPRs) and β-oxidation (Wasternack, 2007). Although the biosynthetic pathways and functions of fungal jasmonates remain elusive, it is suggested that the JA biosynthetic pathway between plants and fungi may be similar (Brodhun et al., 2013). In fact, the pathogenic fungus *Lasiodiplodia theobromae* produced OPDA and an exogenous supplement of synthetic linolenic acid led to jasmonate production (Tsukada et al., 2010), although the presence of JA biosynthetic enzymes (like LOX and AOS) in this fungus was not confirmed (Brodhun et al., 2013). Remarkably, a study characterizing the enzymatic properties of a putative LOX in *F. oxysporum* f. sp. *lycopercisi*, showed that it was similar to plant LOXs, involving iron instead of manganese at the active site (Brodhun et al., 2013).

Following on the hypothesis that JA produced by the fungus could play an important role in overcoming the plant defense, we hypothesized that total or partial silencing of the *OPR* gene could have an effect on the ability of *F. oxysporum* to infect the plant. Our results unambiguously demonstrate that the level of silencing achieved by HD-RNAi in our experiments was sufficient to significantly delay the progression of the disease. Although all genotypes experienced severe stress upon infection with the pathogen, the transgenic lines displayed greatly reduced disease symptoms and a much higher survival rate compared to wild type controls. We suggest that the *OPR*-RNAi transgenic plants were better equipped to withstand the pathogen probably due to down-regulation of the *F. oxysporum OPR* gene and subsequent reduction in jasmonate synthesis by the attacking fungus.

In summary, our results show that HD-RNAi can effectively down-regulate endogenous genes in pathogenic hemi-biotrophs although the degree of silencing is variable depending on the gene targeted and the transgenic line examined. No silencing was observed in a sequence-unrelated fungal gene (Supplementary Figure S3). No complete silencing has been achieved but partial silencing was enough to at least delay the disease progression and increase the rate of survival. HD-RNAi in the Arabidopsis-*F. oxysporum* interaction system could be used to quickly test fungal genes suspected to be involved in pathogenicity. In agricultural terms, achieving a delay in disease progression can have a very practical application as it provides the farmers with extra time to diagnose and treat the pathogen, minimizing crop losses. Furthermore, production of HD-RNAi constructs targeting multiple virulence-related genes should provide more durable resistance than methods relying on a single gene.

### Conflict of interest statement

The authors declare that the research was conducted in the absence of any commercial or financial relationships that could be construed as a potential conflict of interest.
