# Supplementary material for: Down-regulation of Fusarium oxysporum endogenous genes by Host-Delivered RNA interference enhances disease resistance
Source: Front Chem. 2015 Jan 20;3:1. doi: 10.3389/fchem.2015.00001 (PMC4299518; doi:10.3389/fchem.2015.00001)
Supplement: Supplementary file 1 [file Image1.PDF]

SUPPLEMENTARY FIGURE S1

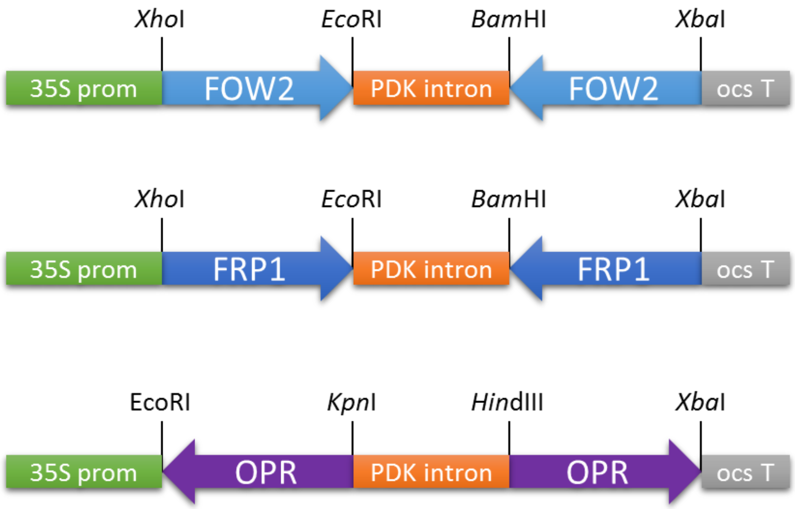

Supplementary Fig. S1. RNAi constructs used in the production of the *FPR1*, *FOW2* and *OPR* RNAi lines. Gene fragments were cloned into the pHannibal or pKannibal RNAi vectors in sense and antisense orientation under the control of cauliflower mosaic virus *35S* and the *OCS*-terminator. The RNAi cassette was later excised and cloned into an appropriate binary vector (for details see **Materials and methods**).
