# Supplementary material for: Down-regulation of Fusarium oxysporum endogenous genes by Host-Delivered RNA interference enhances disease resistance
Source: Front Chem. 2015 Jan 20;3:1. doi: 10.3389/fchem.2015.00001 (PMC4299518; doi:10.3389/fchem.2015.00001)
Supplement: Supplementary file 2 [file Image2.PDF]

SUPPLEMENTARY FIGURE S2

A) CLUSTAL O(1.2.1) alignment of Arabidopsis OPR3 and *F. oxysporum* OPR genes; sequence used for RNAi construct is highlighted in yellow

```
FoOPR        ATGGGCGATATCATCGATG--CCGGAGCTTCTTCCAAGCTCTTCGCTCCTCTGGACATTG
AtOPR3       -------ATGACGGCGGCACAAGGGAACTCTAACGAGACTCTG-----TTTTCTTCTTAC
                    **  *  **        *** **   * *   ****      * *   * *

FoOPR        GAAATGGCAAGATTCAC-CTGAAGCACCGCATTGTTCATGCACCTCTTACCAGAAACCGA
AtOPR3       AAGATGGGAAGATTCGATCTCTCTCATCGAGTGGTTCTGGCGCCGATGA-----------
              * **** *******   **    ** **  * ****  ** **  * *

FoOPR        GGAACTCCGGTCAATACTGAGTCTACGCCGGAGAATCCCAACCGACTCTGGGTTCCCAAT
AtOPR3       ----------------------------CGCGGTGCAGGGCGTTGAACGGAGTACCAAAC
                                         **  *              * * ** ** **

FoOPR        GATCTGATGGCCGAATACTATTCTCAACGAGCGACCGATGGTGGTCTCATCATTTCTGAG
AtOPR3       GCGGCGTTGGCAGAGTATTATGCTCAACGGACCACTCCCGGCGGTTTTCTCATCTCCGAA
             *    * **** ** ** *** *******  * **    ** *** *  **** ** **

FoOPR        GGTCTCCCTCCTTCACTTGAGGGTAATGGAATGCCAGGCGTTCCAGGCATTTTCTTGCCT
AtOPR3       GGCACCATGGTCTCTCCCGGATCCGCAGGGTTCCCACATGTGCCTGGAATCTATTCAGAT
             **   *      ** *  *        **  * ***   ** ** ** ** *  *    *

FoOPR        CAACAGGTAGAAGGCTGGAAGAAGGTCGTTTCTGCTGTTCATTCCAAAGGAGGTTATTTC
AtOPR3       GAACAAGTAGAAGCATGGAAGCAAGTTGTGGAAGCAGTTCACGCTAAGGGAGGTTTCATC
              **** *******  ****** * ** **    ** *****  * ** *******   **

FoOPR        TACGCCCAGCTTTGGCACTCCGGCCGCGCCAACATCCCTCAGTTG------ACTGGAA--
AtOPR3       TTTTGTCAATTATGGCATGTTGGACGTGCTTCTCATGCAGTGTATCAACCTAATGGAGGA
             *     **  * *****    ** ** **        *   **        * ****

FoOPR        -CTCCTATTCTTGCTCCCTCCGCCACACCGTGGGATGACCCTAACGAAACCTTCTCATAT
AtOPR3       TCACCAATATCGTCAACGAACAAACCAATCTCGGAAAACAGGT--GGCGAGTTTTGTTGC
              * ** **     *  *   *    **   * ***  **      *     ** *  *

FoOPR        CCCCCACCGCACAGCACCACGCCCGTCAAAATCGCAGACTATCCGCCCATCGAGATGACC
AtOPR3       CCGAT----------GGTTCCCACGTGAAGTACCCGAAACCTCGGGCTTTAGA------A
             **                 * * *** **   * *  *   ** * *  * **

FoOPR        GTGGATAAGATCAAGAGTACTATCAGTGACTACGTCAACGCCGCAAAGAATGCCGTTGAA
AtOPR3       GCTTCCGAGATACCTCGGGTGGTGGAGGATTATTGCCTTTCTGCTTTGAATGCGATTCGA
             *      ****     *     *    ** **   *    * **   ******  **  *

FoOPR        GCTGGTTTCGATGGCGTTGAAGTCCATGGTGGAAACGGATACCTTGTCGAGCAGTTTCTC
AtOPR3       GCTGGTTTCGATGGGATTGAGATCCACGGGGCGCATGGTTACCTCATTGATCAGTTTTTG
             **************  ****  **** ** *   * ** *****  * ** ****** *

FoOPR        TCTTCAAACATCAACAAACGAACAGACCACTATGGCGGTTCCGAGGAGAAGAGATGCAAC
AtOPR3       AAAGACGGGATCAATGACCGTACTGACCAATACGGAGGATCCATTGAAAACCGTTGTAGA
                      *****  * ** ** ***** ** ** ** ***   ** **  * ** *

FoOPR        TTTGCACTCGAACTTATGGAGGAACTAGCCAAGGCGATCGGTCAAGAGAACCTGGCTATT
AtOPR3       TTCTTGAAACAAGTAGTGGAAGGTGTAGTTTCAGCCATAGGAGCTAGTAAAGTTGGTGTG
             **        ** *  **** *   ***     ** ** **       **  * * * *

FoOPR        CGTCTCACCC--C-TTTTGGTCTGTTTAACCAGGCTCGTG----------GTGAAAAGCG
AtOPR3       AGGGTATCTCCAGCTATAGATCACTTGGACGCAACTGATTCTAACCCATTATCACTCGGG
              *  *  * *    * * * **  **  **    **  *            * *   * *
```

```
FoOPR        CGTTGAAACGTGGAGCT---ACCTCTGCTGTGAGCTGAAATCCCGCTTGCCCAGGCTTTC
AtOPR3       CTAGCCGTGGTTGATATGCTCAATAAGTTACAAGATGTTAATGGCTT----GAAGCTCGC
             *        ** **  *      *  * *   ** **  *      *     * ***  *

FoOPR        GTACGTTAGTTTCATTGAACCGCGGTATGAGCAGATCTTTTCGGAGGAGGAG-----AAA
AtOPR3       TTACCTTCACGTTACACAACCTCGCTACCACGC---CTACGGGCAAACAGAGTCGGGAAG
              *** **    * *   **** ** **  *      **    * *    ***     **

FoOPR        CAAAAGTTCTTGGGATCTTGGGGTTTACCTACCGTCGATCTGTCGCTGTTCCGTTCGATC
AtOPR3       GCAAGGGAGTGATGAGGAAGAAGCTAAGCTAATGAAGAGC-----------TTGAGAATG
               ** *   *   **    *  * * * ***  *  ** *                 **

FoOPR        TTTGGAGATACGCCATTCTTTTCAGCTGGCGGATTCAATGATGTTAACTCCTGGGGTGTT
AtOPR3       GCTTATAAAGGAACCTTTATGTCCAGTGGAGGATTCAATAAGGAACTAGGCATGCAAGCT
               *    *     * **  * **   *** ********* * *       *  *   * *

FoOPR        GTAGAGAGTGGAAAATATGATGCCCTTTTGTATGGAAGGTATTTTATCAGTAACCCTGAT
AtOPR3       GTTCAACAAGGTGATGCTGATTTGGTTTCATATGGCAGGCTTTTTATCGCAAACCCGGAT
             **  *    **  *   ****    ***  ***** ***  *******   ***** ***

FoOPR        TTGGTGGAGAGATTGAGAAACGGTTGGCCGTTGCAGGCATATGATAGGAAGACGTTTTAT
AtOPR3       TTGGTTTCGCGGTTCAAGATTGATGGAAAGTTGAATAAATATAATCGGAAGACGTTTTAC
             *****   * * ** *  *  * * *   **** *   **** ** *************

FoOPR        GGGCCTTTTGAGGATAATGCTGTTGGATATACGGATTATCCCTTTTACAAAAAGGAGTAG
AtOPR3       ACTCAAGATCC------AGTTGTTGGCTACACGGATTATCCTTTCTTGGCTCCTTCTTCC
                *    *         * ****** ** *********** ** *           *

FoOPR        ---------
AtOPR3       CGCCTCTGA
```

B) CLUSTAL O(1.2.1) alignment of Arabidopsis OPR3 and *F. oxysporum* OPR proteins

```
FoOPR        MGDIIDAGASSKLFTPLDIGNGKIHLKHRIVHAPLTRNRGTPVNTESTPENPNRLWVPND
AtOPR3       -MTAAQGNSNETLFSSY--KMGRFDLSHRVVLAPMTRCRAL-------------NGVPNA
                  :. :...**:      *::.*.**:* **:** *.                ***

FoOPR        LMAEYYSQRATDGGLIISEGLPPSLEGNGMPGVPGIFLPQQVEGWKKVVSAVHSKGGYFY
AtOPR3       ALAEYYAQRTTPGGFLISEGTMVSPGSAGFPHVPGIYSDEQVEAWKQVVEAVHAKGGFIF
              :****:**:* **::****   *  . *:* ****:  :***.**:**.***:***:::

FoOPR        AQLWHSGRANIPQLT---GTPILAPS----ATPWDD---PNETFSYPPPHSTTPVKIADY
AtOPR3       CQLWHVGRASHAVYQPNGGSPISSTNKPISENRWRVLLPDGSHVKYPK------------
             .**** ***.        *:** : .     . *       . ..**

FoOPR        PPIEMTVDKIKSTISDYVNAAKNAIEAGFDGVEVHGGNGYLVEQFLSSNVNKRTDHYGGS
AtOPR3       -PRALEASEIPRVVEDYCLSALNAIRAGFDGIEIHGAHGYLIDQFLKDGINDRTDQYGGS
              *  : ..:*  .:.**  :* ***.*****:*:**..***::***.. :*.***:****

FoOPR        EEKRCNFAIELMEELAKAIGQENLAIRLTPFGLFNQSRGEKRVE----TWSHLCRELKSR
AtOPR3       IENRCRFLKQVVEGVVSAIGASKVGVRVSPAIDHLDATDSNPLSLGLAVVDMLNKLQDVN
              *:**.*  :::* :..*** .::.:*::*   . ::  .: :.    . . * :  . .

FoOPR        LPKLSYVSFVEPRYEQIFSEEEKQKFLGSWGLPTVDLSLFR--SIFGDTPFFSAGGFNDV
AtOPR3       GLKLAYLHVTQPRYHAYGQTESG-----RQGSDEEEAKLMKSLRMAYKGTFMSSGGFNKE
               **:*: ..:***.   . *.        *    : .*::   :  .  *:*:****.

FoOPR        NSWGVVESGKYDALLYGRYFISNPDLVERLRNGWPLQAYDRKTFYGPFEDNAVGYTDYPF
```

```
AtOPR3      LGMQAVQQGDADLVSYGRLFIANPDLVSRFKIDGKLNKYNRKTFYT--QDPVVGYTDYPF
             .   .*:.*. * : *** **:*****.*::    *: *:*****   :* .********

FoOPR       YKKE---
AtOPR3      LAPSSRL
           .
```
