# Supplementary figures and images for: Down-regulation of Fusarium oxysporum endogenous genes by Host-Delivered RNA interference enhances disease resistance
Source: Front Chem. 2015 Jan 20;3:1. doi: 10.3389/fchem.2015.00001 (PMC4299518; doi:10.3389/fchem.2015.00001)

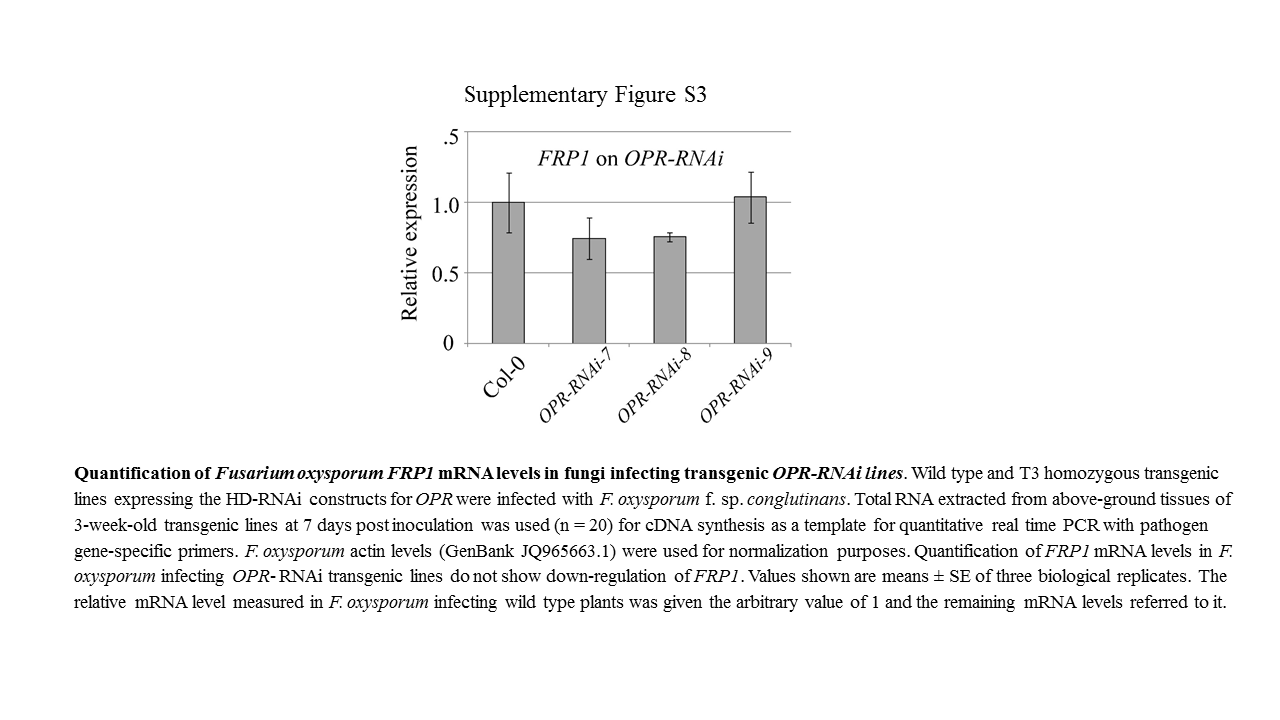

Supplement: Supplementary file 3 [file Image3.TIF]
